# Isolated Nail Lichen Planus With Extensive Involvement: The Importance of Clinicopathologic Correlation and Response to Hydroxychloroquine

**DOI:** 10.7759/cureus.114948

**Published:** 2026-08-21

**Authors:** Bryan Salvador Sol, Diana Maria Ibarra Tostado, Andrea Sierra, Alejandra Hernandez, Andrea Zaragoza Navarro, Teresa Jannete Ortega Valls, Maria Elena Reyes Moreno

**Affiliations:** 1 Dermatology, Instituto Dermatológico de Jalisco "Dr. José Barba Rubio", Zapopan, MEX

**Keywords:** hydroxychloroquine treatment, nail biopsy, nail lichen planus, onychodystrophy, onychoscopy

## Abstract

Nail lichen planus is an uncommon inflammatory disorder that can occur as an isolated condition and may lead to permanent nail scarring when diagnosis is delayed. We present the case of a 50-year-old woman with progressive dystrophy affecting 16 fingernails and toenails, associated with pain and impaired ambulation. Clinical examination and onychoscopy raised suspicion of nail lichen planus, while repeated mycological studies were negative. Histopathological examination of a nail matrix biopsy confirmed the diagnosis. Treatment with systemic prednisone did not result in clinical improvement. Subsequently, hydroxychloroquine (200 mg/day) combined with topical 0.05% clobetasol led to progressive improvement over 12 months, characterized by decreased pain, reduced periungual inflammation, and growth of less dystrophic nail plates without evidence of further irreversible damage. This case emphasizes the importance of considering nail lichen planus in patients with multifocal nail dystrophy and negative mycological studies. Early clinicopathological correlation is essential for diagnosis and timely management. Hydroxychloroquine combined with topical clobetasol may be considered as a therapeutic approach in selected patients with inadequate response to systemic corticosteroids.

## Introduction

Nail lichen planus is an uncommon variant of lichen planus that affects the nail unit and represents one of the major inflammatory causes of onychodystrophy associated with a high risk of permanent scarring. Although nail involvement occurs in approximately 10% of patients with lichen planus, it may present as the sole manifestation of the disease in a substantial proportion of cases, making recognition challenging and leading to diagnostic delays that increase the risk of irreversible nail matrix destruction [1].

Since its initial description in 1970, understanding of this entity has advanced considerably, allowing better recognition of the relationship between the anatomical site involved and the characteristic nail changes [2]. Subsequently, longitudinal nail unit biopsy became established as the diagnostic gold standard in atypical presentations or when differentiation from other causes of nail dystrophy remains uncertain [3,4]. More recently, onychoscopy has enabled the identification of additional findings, such as distal nail plate fraying, which complements the clinical evaluation without replacing histopathological examination [5].

The management of nail lichen planus remains challenging because of the lack of comparative studies and the absence of disease-specific clinical trials. Although corticosteroids remain the cornerstone of first-line therapy, their long-term use may be limited by adverse effects, disease relapse, and the need for maintenance treatment. In this context, hydroxychloroquine has emerged as an immunomodulatory alternative with a favorable safety profile and a biologically plausible mechanism of action for T-cell-mediated inflammatory diseases. In the present case, hydroxychloroquine was selected over other systemic immunosuppressive options because of its immunomodulatory properties, relatively favorable long-term safety profile, and potential suitability for maintenance therapy in a chronic disease with extensive nail involvement. However, evidence supporting its use in nail lichen planus remains limited and is largely restricted to case reports and small case series [1,6].

We report the case of a patient with isolated nail lichen planus with extensive nail involvement, confirmed through clinicopathological correlation, who achieved a favorable clinical outcome following treatment with hydroxychloroquine after an insufficient response to systemic corticosteroids.

## Case presentation

A 50-year-old woman with no significant past medical history presented with progressively worsening nail changes. The disease initially manifested after an eight-month course with nail thickening (onychauxis) and xanthonychia affecting the left great toenail, subsequently progressing to involve a total of 16 nail plates. Clinical examination revealed onychauxis, trachyonychia, longitudinal ridging, and xantholeukonychia affecting the involved nails, accompanied by periungual erythema and edema in some digits (Figure 1).

**Figure 1:**
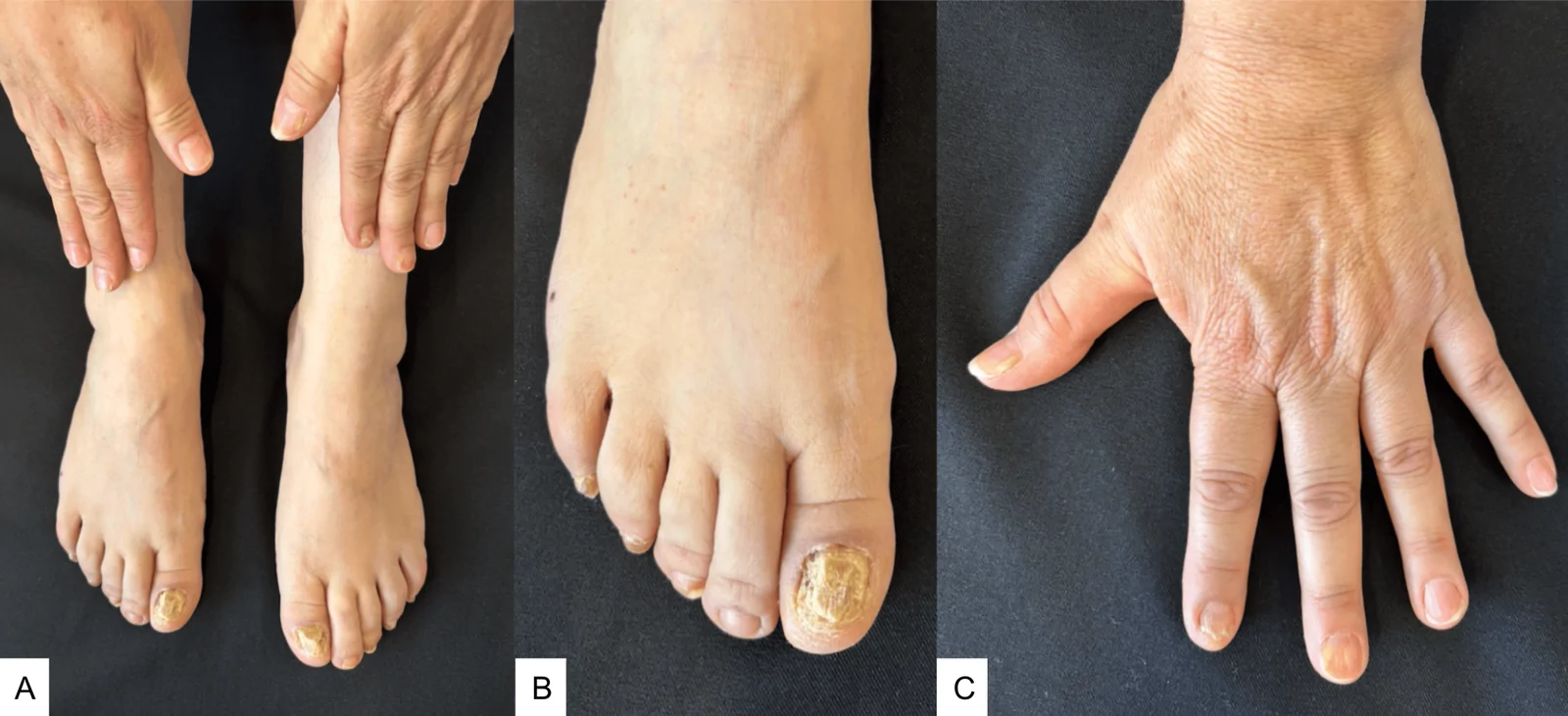
Baseline clinical presentation of nail involvement. (A) Macroscopic overview of all affected fingernails and toenails. (B) Clinical appearance of the affected toenails demonstrating onychauxis, trachyonychia, longitudinal ridging, xantholeukonychia, and periungual inflammation. (C) Clinical appearance of the affected fingernails showing similar dystrophic changes

The patient reported pain with ambulation and tenderness to minimal pressure, with an apparently chronic course.

Dry and immersion onychoscopy demonstrated splinter hemorrhages, onychorrhexis, subungual erythema, xanthonychia, and distal nail plate fraying (Figure 2).

**Figure 2:**
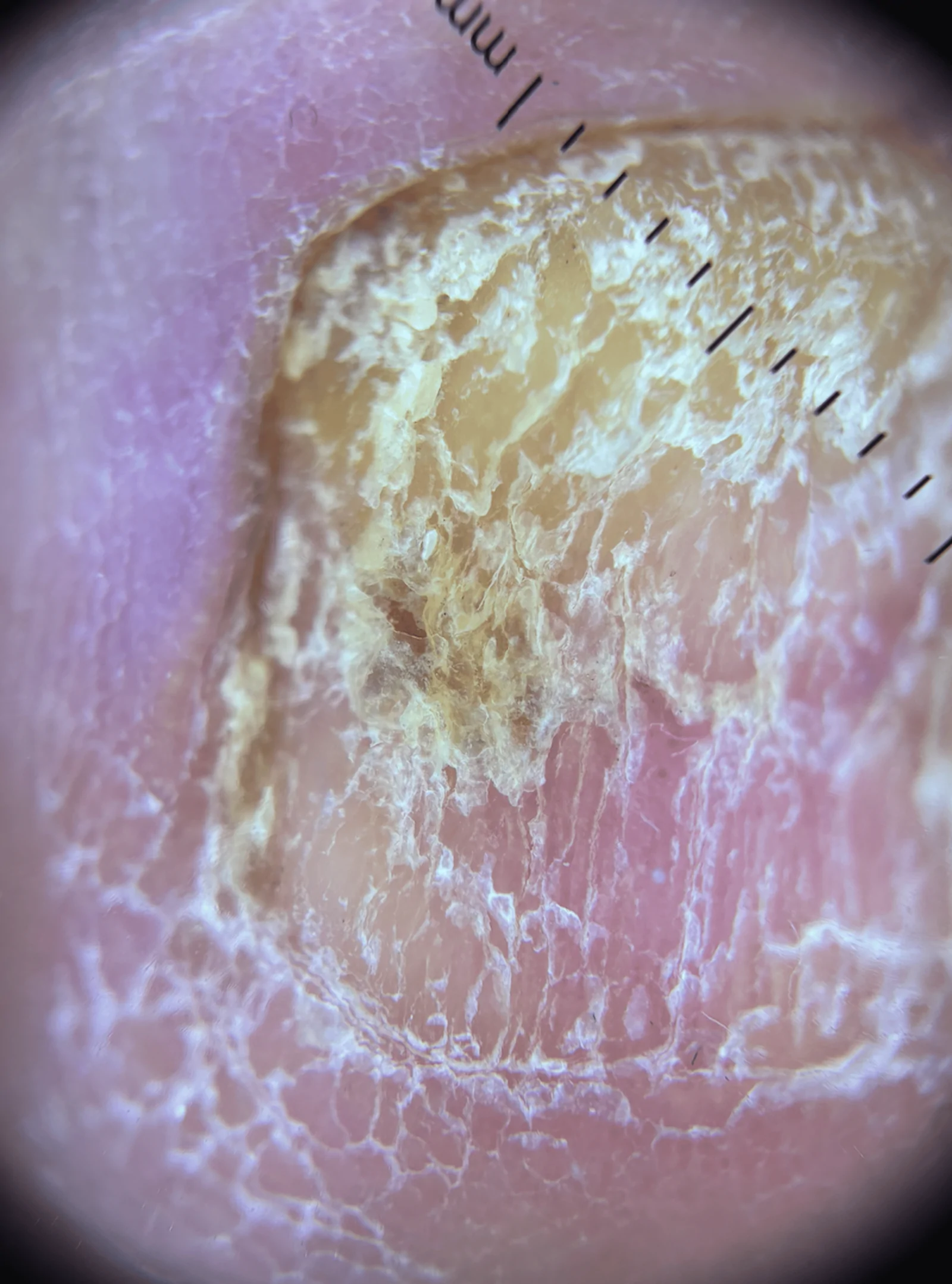
Onychoscopic examination of an affected toenail demonstrating longitudinal ridging, onychorrhexis, splinter hemorrhages, xanthonychia, and distal nail plate fraying

Given the suspicion of onychomycosis, three mycological examinations were performed at different time points, all of which yielded negative results. A 3.5-mm punch biopsy of the distal nail matrix was subsequently obtained for histopathological evaluation with hematoxylin and eosin staining. Histopathological examination revealed compact hyperkeratosis with orthokeratosis, hypergranulosis, acanthosis, sawtooth elongation of the rete ridges, and vacuolar degeneration of the basal layer. The dermis showed a band-like lymphocytic inflammatory infiltrate. No fungal elements were identified (Figure 3).

**Figure 3:**
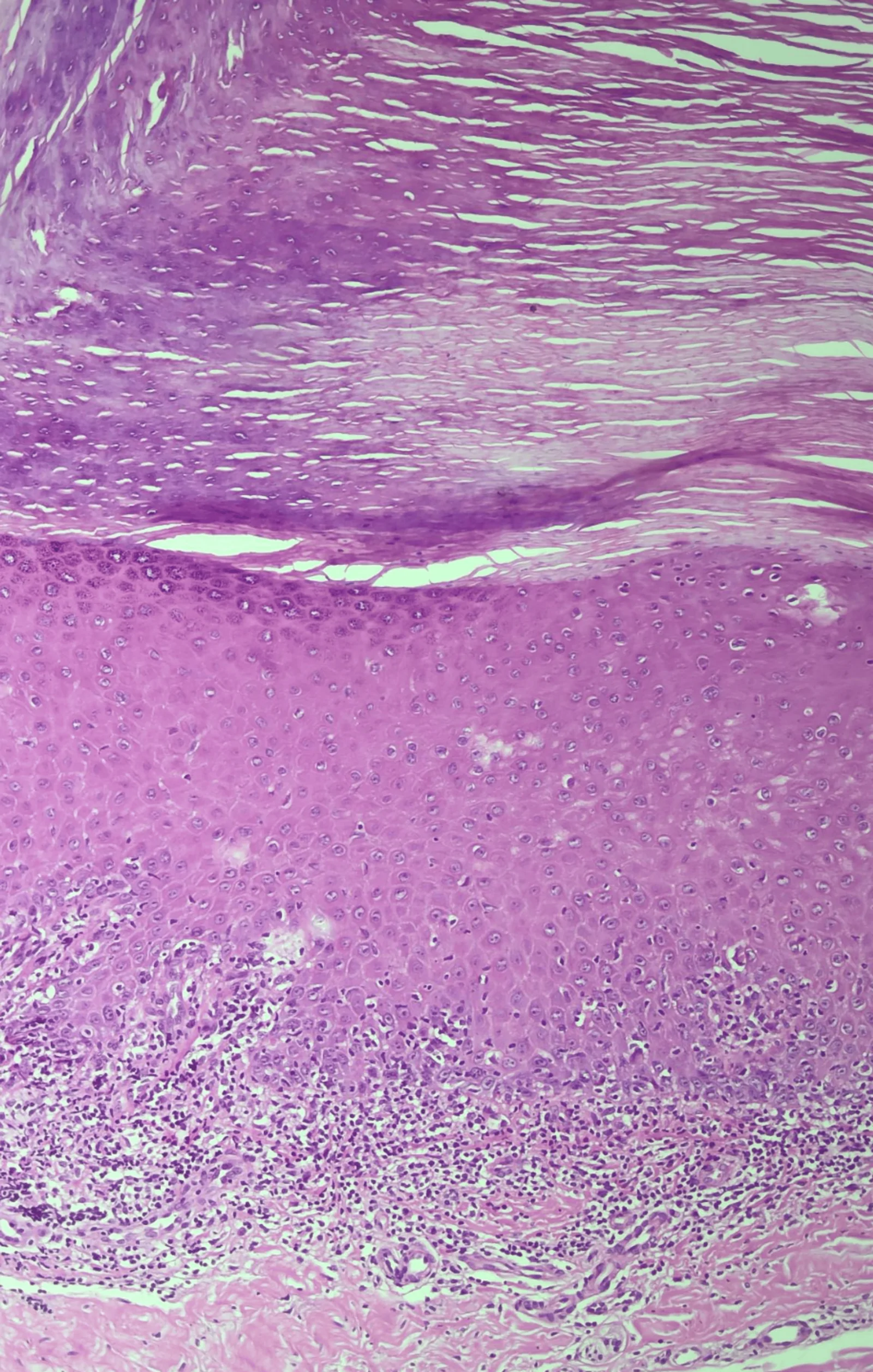
Histopathological examination of the distal nail matrix (hematoxylin and eosin stain, 40× magnification) showing compact orthokeratotic hyperkeratosis, hypergranulosis, irregular acanthosis with sawtooth elongation of the rete ridges, basal vacuolar degeneration, and a band-like lymphocytic inflammatory infiltrate, consistent with lichenoid interface dermatitis

Based on the clinicopathological correlation, a diagnosis of isolated nail lichen planus was established. Initial treatment with systemic prednisone (50 mg daily for four weeks) failed to produce clinical improvement. Hydroxychloroquine was subsequently initiated in combination with topical 0.05% clobetasol, resulting in marked clinical improvement across all affected nail plates (Figure 4).

**Figure 4:**
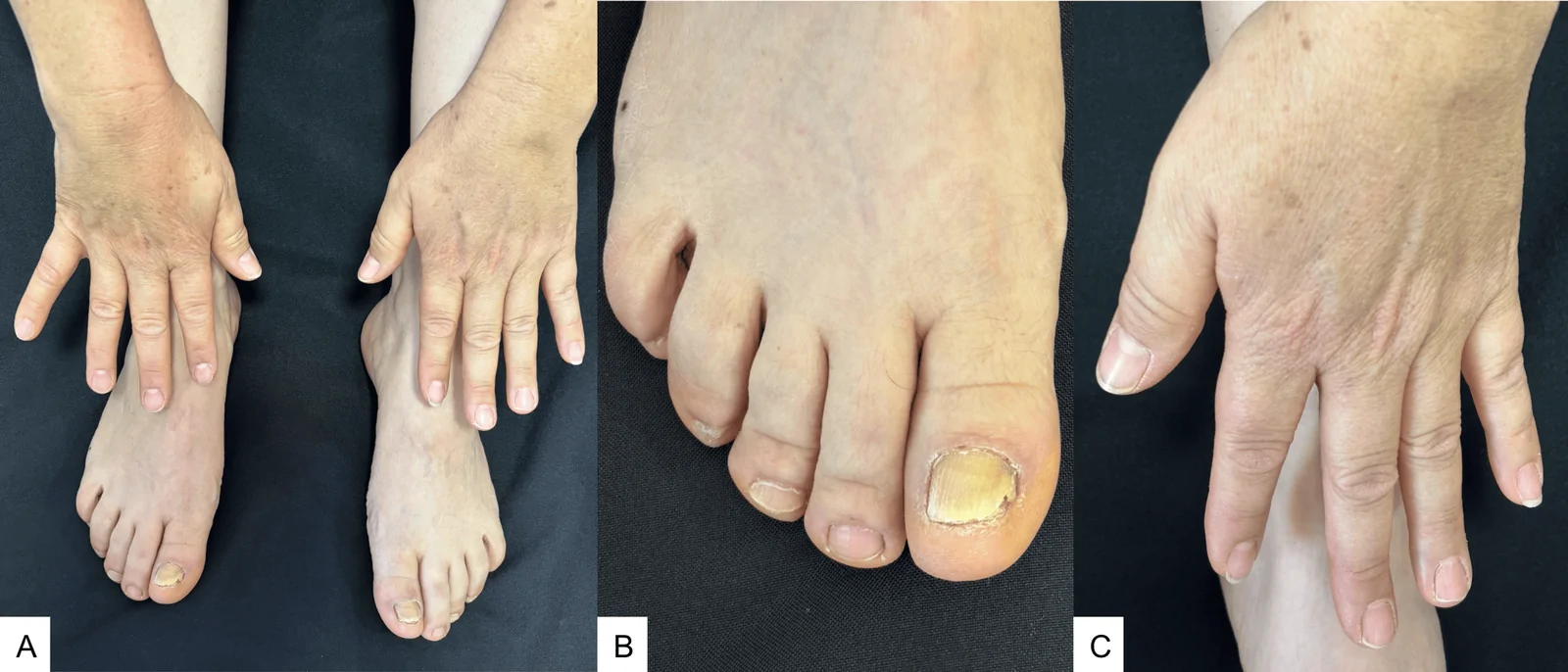
Clinical improvement after 12 months of treatment with hydroxychloroquine and topical 0.05% clobetasol. (A) Macroscopic overview of the fingernails and toenails demonstrating proximal growth of less dystrophic nail plates. (B) Close-up view of the affected toenails showing reduced periungual inflammation and improvement in nail plate morphology. (C) Close-up view of the affected fingernails demonstrating similar clinical improvement

## Discussion

Nail lichen planus is an uncommon manifestation of lichen planus that may involve the nail matrix, nail bed, and periungual tissues. Nail involvement occurs in approximately 10-15% of patients with lichen planus but may also present as an isolated disease without concomitant cutaneous, hair, or mucosal involvement [1,2]. This isolated presentation poses a diagnostic challenge because the absence of the classic manifestations of lichen planus may delay diagnosis and increase the risk of irreversible scarring, dorsal pterygium, nail atrophy, or anonychia [2].

Our patient presented with an isolated, bilateral, symmetrical, and extensive form of the disease involving 16 fingernails and toenails. Longitudinal ridging and fissuring, onychorrhexis, trachyonychia, xanthonychia, and periungual inflammation have all been previously described in nail lichen planus [2,3]. Simultaneous involvement of both the hands and feet, together with pain on pressure and impaired ambulation, reflected clinically significant disease despite the absence of dorsal pterygium or anonychia. In this setting, preservation of the nail plate and the absence of advanced scarring provided an opportunity to initiate treatment before permanent structural damage occurred.

The main differential diagnoses included onychomycosis and nail psoriasis. Onychomycosis may present with xanthonychia, onychodystrophy, subungual hyperkeratosis, and onycholysis, whereas nail psoriasis commonly manifests with onycholysis, subungual hyperkeratosis, splinter hemorrhages, and nail plate abnormalities [4]. In our patient, the absence of nail pitting and oil-drop discoloration made psoriasis less likely, whereas three negative mycological examinations and fungal cultures, together with the lack of response to topical ciclopirox, argued against a fungal infection. Nevertheless, these findings alone were insufficient to establish the diagnosis, making clinicopathological correlation with onychoscopic and histopathological findings essential.

Onychoscopy is a useful noninvasive tool for identifying abnormalities of the nail matrix and nail bed, selecting the optimal biopsy site, and recognizing early or clinically ambiguous cases. Reported onychoscopic features of nail lichen planus include longitudinal ridging, onychorrhexis, splinter hemorrhages, erythema or lunular abnormalities, onycholysis, nail discoloration, and nail plate fragmentation [3,5]. In our patient, the presence of longitudinal ridging, xanthonychia, onychorrhexis, splinter hemorrhages, and subungual erythema supported the clinical suspicion.

Another notable finding was distal nail plate fraying. This onychoscopic feature is characterized by irregular fissuring or fragmentation of the free edge and has recently been described in patients with nail lichen planus [3,5]. Although it is neither pathognomonic nor predictive of progression to dorsal pterygium, it may serve as an additional clue to chronic longitudinal nail plate damage and reinforce the need for early diagnostic evaluation.

Histopathological examination remains essential when clinical and onychoscopic findings do not adequately distinguish nail lichen planus from other causes of nail dystrophy. The biopsy site should be selected according to the predominantly affected nail structure, as nail plate abnormalities generally reflect matrix disease, whereas onycholysis, subungual hyperkeratosis, and certain nail discolorations are more suggestive of nail bed involvement [2,6]. In our patient, distal nail matrix biopsy demonstrated orthokeratotic hyperkeratosis, hypergranulosis, irregular acanthosis, basal vacuolar degeneration, and a band-like lymphocytic infiltrate, findings consistent with lichenoid interface dermatitis [6]. The absence of Civatte bodies did not exclude the diagnosis, as the histopathological features of nail lichen planus may vary according to both the biopsy site and the stage of disease [6].

Nail lichen planus is considered a relative therapeutic emergency because of its potential to cause permanent matrix scarring. Expert consensus recommends intralesional or intramuscular corticosteroids, particularly triamcinolone acetonide, as first-line therapy, whereas systemic retinoids and other immunomodulatory agents may be considered in extensive, progressive, or treatment-resistant disease [2]. However, current therapeutic recommendations are largely based on retrospective studies, case reports, and expert opinion, as adequately powered randomized controlled trials are lacking [2,7].

In our patient, involvement of 14 nails, disabling pain, and impaired ambulation justified the initiation of systemic therapy. Prednisone was administered for four weeks without meaningful clinical improvement. Because of the inadequate response and the patient's preference to avoid prolonged systemic corticosteroid therapy, treatment was switched to hydroxychloroquine in combination with topical clobetasol.

Evidence supporting the use of hydroxychloroquine in nail lichen planus remains limited, and the drug is not currently recommended as first-line therapy in expert consensus statements [2,7]. Although its immunomodulatory effects provide a biologically plausible rationale for use in T-cell-mediated inflammatory diseases, published outcomes in nail lichen planus have been inconsistent. In the largest retrospective series of 81 biopsy-confirmed patients, 62 had treatment follow-up, including four patients treated with hydroxychloroquine; three had moderate disease, one had severe disease, and none demonstrated meaningful clinical improvement [8]. This contrasts with the favorable response observed in our patient, who achieved clinical improvement after an inadequate response to systemic corticosteroids. Given the small number of patients previously treated with hydroxychloroquine and the retrospective nature of the available evidence, these findings should be interpreted cautiously. Our case therefore represents an additional observation contributing to the limited literature on hydroxychloroquine as a potential therapeutic option for nail lichen planus, rather than conclusive evidence of its efficacy.

After 12 months of treatment with hydroxychloroquine (200 mg/day) and topical clobetasol 0.05%, the patient experienced sustained improvement in pain and periungual inflammation, recovery of normal ambulation, and proximal growth of less dystrophic nail plates. No adverse effects or progression to dorsal pterygium or anonychia were observed. However, because topical clobetasol was administered concomitantly, the clinical response cannot be attributed solely to hydroxychloroquine. Furthermore, the inherent limitations of a single case report preclude conclusions regarding causality or generalizability.

The principal strength of this report lies in the clinicopathological correlation of an isolated and extensive presentation supported by characteristic onychoscopic findings and histopathological confirmation. In addition, it documents sustained clinical improvement over 12 months following failure of an initial course of systemic corticosteroids. This case underscores the importance of considering nail lichen planus in patients with multifocal nail dystrophy and negative mycological studies, even in the absence of cutaneous, mucosal, or scalp manifestations of lichen planus. Early recognition, targeted biopsy, and prompt initiation of treatment remain essential to preserve nail matrix function and prevent irreversible nail sequelae.

## Conclusions

Nail lichen planus should be considered in patients presenting with progressive dystrophy involving multiple nails and negative mycological studies, even in the absence of cutaneous, hair, or mucosal manifestations. In this case, the correlation of clinical, onychoscopic, and histopathological findings enabled an accurate diagnosis before the development of irreversible nail scarring.

The sustained clinical improvement observed with hydroxychloroquine in combination with topical clobetasol, following an inadequate response to systemic prednisone, suggests a potential therapeutic option for selected patients with extensive disease. However, this favorable outcome should not be interpreted as evidence of treatment efficacy and warrants further evaluation in larger studies. Early recognition, targeted nail biopsy, and timely treatment remain essential to preserve nail matrix function and prevent irreversible nail sequelae.
